# The effects of food and parasitism on reproductive performance of a wild rodent

**DOI:** 10.1002/ece3.3997

**Published:** 2018-03-26

**Authors:** Pei‐Jen L. Shaner, Ai‐Yun Yu, Shou‐Hsien Li, Ching‐Ho Hou

**Affiliations:** ^1^ Department of Life Science National Taiwan Normal University Taipei Taiwan

**Keywords:** breeding, diseases, fitness, host‐parasite, life history, nematode

## Abstract

Food and parasitism can have complex effects on small mammal reproduction. In this study, we tested the effects of sex, food, and parasitism on reproductive performance of the Taiwan field mouse (*Apodemus semotus*). In a field experiment, we increased food availability for a portion of the mice in the population by providing sorghum seeds to a set of food stations. We reduced parasite intensity of randomly chosen mice through ivermectin treatment. We determined the number and quality of offspring for the mice using paternity analysis. We quantified seed consumption with stable carbon isotope values of mouse plasma and parasite intensity with fecal egg counts of intestinal nematodes and cestodes (FEC). In a laboratory experiment, we reduced parasite intensity of randomly chosen mice through ivermectin treatment. We quantified their immune functions by total white blood cell count, percent granulocyte count, and percent lymphocyte count through hematological analyses. We measured the FEC and energy intake of the mice. From the field experiment, the number of offspring in *A. semotus* increased with increasing seed consumption. Due to the trade‐off between number and quality of offspring, the offspring quality decreased with increasing seed consumption for the females. The ivermectin treatment did not affect offspring number or quality. However, the FEC was positively correlated with number of offspring. In the laboratory experiment, the percent lymphocyte/granulocyte count changed with parasite intensity at low energy intake, which was relaxed at high energy intake. This study demonstrated positive effects of food availability and neutral effects of parasitism on *A. semotus* reproduction. However, the benefits of food availability for the females need to take into account the offspring number–quality trade‐off, and at high infection intensity, parasitism might negatively affect offspring quality for the males. We suggest that food availability could mediate the relationships between parasite intensity and immune responses.

## 1 INTRODUCTION

Reproduction is a key component of life history and population dynamics. In small mammals, reproductive performance is typically constrained by food availability (reviewed in Bronson, 1985; Bronson & Perrigo, 1987; Boutin, 1990; Speakman, 2008). However, other factors such as predation and parasitism may also play a role. Although there have been many studies examining parasitism effects on number and quality of offspring in small mammals, the results are mixed. For instance, despite that several studies found negative parasitism effects on host reproduction in squirrels (e.g., *Xerus inauris*, Hillegass, Waterman, & Roth, 2010; *Tamiasciurus hudsonicus*, Gooderham & Schulte‐Hostedde, 2011; Patterson, Neuhaus, Kutz, & Ruckstuhl, 2013), both negative and neutral effects have been reported for the same host species (*Urocitellus columbianus*, Neuhaus, 2003; Raveh et al., 2011; Raveh, Neuhaus, & Dobson, 2015). Therefore, our understanding of parasitism effects on mammalian reproduction remains controversial.

Reproductive performance can be studied at population or individual level. At population level, food supplementation or parasite removal has been found to increase the proportion of breeding individuals and allow breeding during seasonal reproductive hiatus (e.g., food supplementation, Banks & Dickman, 2000; Forbes, Stuart, Mappes, Henttonen, & Huitu, 2014; parasite removal, Vandegrift, Raffel, & Hudson, 2008; but see Forbes et al., 2014). However, due to differential competitive abilities, individuals within a population may respond differently to increased food availability or reduced parasitism. These individual‐level responses can reveal patterns in microevolutionary processes such as life‐history trade‐offs and phenotypic shifts (Britton & Andreou, 2016; Moore & Wilson, 2002; Sheldon & Verhulst, 1996). In wild, it is often necessary to perform parentage analysis on a breeding population to quantify individual reproductive performance. A breeding population can be substantially larger in area size than a population used for demographic studies even for small mammals with limited mobility (e.g., parentage studies: 0.5–23 ha, García‐Navas, Bonnet, Waldvogel, Camenisch, & Postma, 2016; Gooderham & Schulte‐Hostedde, 2011; Patterson & Schulte‐Hostedde, 2011; Shaner, Yu, Ke, & Li, 2017; demographic studies: 0.05–0.5 ha, Forbes et al., 2014; Lo & Shaner, 2015; Pedersen & Greives, 2008; Vandegrift et al., 2008). Furthermore, individual reproductive success mainly reflects relative fitness, which is more meaningful in the context of a breeding population. Therefore, empirical studies on individual reproductive performance in wild sometimes involve just one single population (e.g., Gooderham & Schulte‐Hostedde, 2011; Shaner et al., 2017).

Life‐history theory predicts that organisms allocate resources between survival and reproduction to maximize fitness (Stearns, 1992). Most endoparasites are nonlethal for small mammals (Munger & Karasov, 1989; Schwanz, 2006), but they can still have substantial impacts on their energetics (Scantlebury, Waterman, Hillegass, Speakman, & Bennett, 2007), physiological conditions (Schwanz, 2006), mate choice (Ehman & Scott, 2002), and thereby life‐history trade‐offs (Lochmiller & Deerenberg, 2000; Michalakis & Hochberg, 1994). For example, anthelminthic treatment was found to increase maternal investment at the cost of lowered future survival in wild populations of the Taiwan field mice (*Apodemus semotus*; Lo & Shaner, 2015). In laboratory, female deer mice (*Peromyscus maniculatus*) inoculated with a blood trematode suffered higher mortality but produced heavier offspring (Schwanz, 2008). Therefore, endoparasitism is a potentially important factor in the evolution of host life history (Sheldon & Verhulst, 1996).

Food and parasitism may interact to influence host reproductive performance (Díaz & Alonso, 2003; but see Murray, Keith, & Cary, 1998), density fluctuations of host populations (Forbes et al., 2015; Pedersen & Greives, 2008; but see Forbes et al., 2014), and spatiotemporal dynamics of diseases (Becker, Streicker, & Altizer, 2015; Forbes et al., 2015; Shaner et al., 2017). At individual level, the interactive effects of food and parasitism are often shaped by how a host individual allocates limited resources to reproduction, defenses, and other behavioral or physiological processes that are important for survival (e.g., antipredation behaviors, thermoregulation). Immune responses are costly defenses against parasitism (Sheldon & Verhulst, 1996), which explains why food availability has been found to constrain immune responses to nematode infections in field voles (*Microtus agrestis*, Forbes et al., 2016). Therefore, immune responses are likely involved in the effects of food and parasitism on reproductive performance of host individuals.

In sexually reproducing species, males and females face unique challenges in resource allocation, such as immunity‐testosterone trade‐offs unique for males (Folstad & Karter, 1992; Stoehr & Kokko, 2006) and energetic demands of gestation and lactation unique for females (Speakman, 2008). Furthermore, spacing behaviors could differ between sexes, influencing both their resource acquisition and parasite transmission. For example, female voles (*Microtus californicus*, Ostfeld, 1986; *Clethrionomys rufocanus*, Ims, 1987) became spatially more aggregated than males under food supplementation, and male *A. semotus* became spatially more aggregated than females after they were reduced of nematode infection (Shaner et al., 2017). This is why sex‐specific effects of food and parasitism on host reproduction are well documented in mammals (Díaz & Alonso, 2003; Gooderham & Schulte‐Hostedde, 2011; Lo & Shaner, 2015).

The goal of this study is to test the effects of sex, food availability, and parasite intensity on individual reproductive performance (i.e., number and quality of offspring) in a wild population of the Taiwan field mice (*A. semotus*). In a field experiment on a single population, we used food addition and anthelminthic (ivermectin) treatment to manipulate food availability and parasite intensity, respectively, for individual mice and estimated their reproductive performance using parentage analysis. In a laboratory experiment, we examined the relationships between energy intake, parasite intensity and immune responses of wild‐caught *A. semotus*. We hypothesize that: (1) food availability has positive effects on *A. semotus* reproduction; (2) parasite intensity has negative effects on *A. semotus* reproduction; (3) food availability and parasite intensity interact to influence *A. semotus* reproduction; and (4) energy intakes will modulate the relationship between parasite intensity and immune responses in *A. semotus*. Given the known male‐biased sexual size dimorphism (males *c*. 28 g, females *c*. 23 g; Lo & Shaner, 2015) and high fecundity costs to females (e.g., at a mean litter size of 4, one litter of neonates is equivalent to *c*. 24% of a mother's body mass; Lin & Shiraishi, 1992a, 1993) in this species, we expect to observe sex differences in the effects of food availability and parasite intensity on *A. semotus* reproduction.

## 2 MATERIALS AND METHODS

### 2.1 Study species

*Apodemus semotus* live for a few months to a year in wild (Lin et al., 2014; Lo & Shaner, 2015). The females have 1–2 litters in their lifetime (Huang, Lin, & Alexander, 1997) with a mean litter size of 4 (range: 2–7; Lin & Shiraishi, 1992a; Yu, 1993). Although they are capable of breeding year‐round, breeding activities are higher from spring to autumn and lower in winter (Huang et al., 1997; Lin & Shiraishi, 1992a; Lin et al., 2014; Lo & Shaner, 2015). Their mating system is likely promiscuous (Bryja et al., 2008; also see Results of this study). They are habitat generalists, inhabiting forests, grasslands, and subalpine shrubs (Yu, 1993). The home range size is *c*. 1513–2547 m^2^ (Lin & Shiraishi, 1992b; Shaner et al., 2017) with high site fidelity (seasonal shifts in activity center *c*. 8–25 m; Lin & Shiraishi, 1992b). Although they are solitary, they do not exhibit strong territoriality (26%–59% of home range use is exclusive; Lin & Shiraishi, 1992b; mean number of conspecifics with overlapping home range is 5 and 7 for females and males, respectively; Shaner et al., 2017). The sex differences in the size, stability, and exclusiveness of their home ranges are relatively small, with males having slightly larger and less exclusive home ranges (Lin & Shiraishi, 1992b; Shaner et al., 2017).

### 2.2 Study site and mouse trapping

The study was conducted in an evergreen forest in Taiwan (121°18′ E, 24°21′ N; elevation *c*. 1800 m). The site was on a river terrace isolated from other forested areas by rivers, paved roads, and orchards. The physical isolation of the site from other suitable habitats helped to ensure a high capture rate (*c*. 99%; Hou et al., 2016) that is critical to parentage analysis. A 9‐ha grid comprised of 332 trapping stations (15 m between adjacent stations; Figure S1) was set up to cover the entire site. A capture–recapture study was performed from May to September 2013, coinciding with the breeding season for this *A. semotus* population (Lin et al., 2014). A 3‐day trapping session was performed every 2 weeks. In the evening, two live traps were set up at each trapping station, baited with a chunk of sweet potato and a ball of peanut butter mixed with a variety of seeds and mealworms. We provided a wool ball in the trap to keep the animal warm. The traps were checked in the morning and closed for the day. All captured mice were brought back to the field station for processing, and the traps that showed signs of being used by any animals were replaced with clean ones. Each mouse was marked with a radio‐frequency chip (Watron Technology Corp., Hsinchu, Taiwan) for individual identification. The sex and age (adult or juvenile, based on pelage color and body mass) of the mouse were recorded. The mice were given parasitism reduction treatment (see “*Anthelmintic treatment*”) and had their tissue samples taken (see “*Food addition*” and “*Parentage analysis*”) before they were released at the trapping station where they were captured in the evening on the same day.

### 2.3 Anthelmintic treatment

Upon first capture, a mouse was randomly assigned to either ivermectin (Lambriar Animal Health Care, Fairbury, NE, USA; dosage: 250 μg/μl; Lo & Shaner, 2015) or water (control) treatment. Once assigned, the individual continued to receive the same treatment upon recapture. No individuals received more than one dose of ivermectin or water per month, and 75% of the individuals received between one and two dosages throughout the study. Pedersen and Antonovics (2013) showed that intestinal nematodes can be cleared by ivermectin in the deer mice (*Peromyscus* spp.). However, in their system, the natural prevalence of intestinal nematodes was *c*. 30% and the study lasted only 4 weeks during which reinfection was less likely an issue. In our system, the natural prevalence of intestinal nematodes was >90% (Shaner et al., 2017), and reinfection for both treated and control mice was expected due to the length of the study (5 months). Therefore, even though we were able to clear intestinal nematodes and cestodes for 6 of 68 treated mice, compared to 0 of 74 control mice, the ivermectin treatment in this study should be viewed as a way to reduce infection intensity rather than to remove parasites.

The infection intensity of all nematodes and cestodes (herein “parasite intensity”) was quantified by fecal egg count (FEC, number of nematode and cestode eggs per gram of mouse feces) following Hou et al. (2016). The common parasite taxa included strongyle nematodes, *Strongyloides* spp., *Trichuris* spp., *Syphacia* spp., *Physaloptera* spp., *Ascaris* spp., *Heterakis* spp., and *Hymenolepis* spp. (Shaner et al., 2017). Although FEC can be problematic as a proxy for parasite loads (i.e., the numbers of adult worms in a host individual), it is still a valuable noninvasive way of assessing relative infection intensity across groups of individuals at intraspecific level (Bryan & Kerr, 1989; McKenna, 1981; Patterson & Schulte‐Hostedde, 2011). In this study, the strongyle eggs dominated the egg counts (strongyle eggs/all eggs = 87% ± 1% *SE*; Shaner et al., 2017), and the ivermectin treatment effectively lowered the infection intensity of strongyle nematodes by 50% and 66% for the treated females and males, respectively, compared to their controls (Shaner et al., 2017). For FEC of all nematodes and cestodes, we confirmed a mean reduction of 68% and 69% for the treated females and males, respectively, compared to their controls (Figure S2), and neither the FEC nor ivermectin efficacy differed between sexes (Table S1).

### 2.4 Food addition

We set up 24 food stations that were arranged in three spatial clusters within the study site (Figure S1). Within each cluster, we randomly selected eight trapping stations (>30 m apart from one another) as the locations of the food stations. Between the second and the seventh trapping sessions, 1 kg of sorghum (*Sorghum bicolour*) seeds was provided at each food station every 2 weeks, with the seeds scattered by hand on the ground within *c*. 2 m of the station marker. *Apodemus semotus* is omnivorous, feeding on seeds, insects, and fungi (Kan, 1995; Shaner et al. 2013). The sorghum seeds contain 358 Kcal/100 g with 12.5% protein. The seeds were added at the end of each trapping session, and they were typically gone by the beginning of the following trapping session. We confirmed that the mice did not become spatially aggregated at the food stations by comparing the mean number of *A. semotus* captures at the 24 food stations to a bootstrapped distribution for the mean numbers of captures under the assumption of random space use with respect to the locations of the food stations (*p *=* *.15; Figure S3).

The mice at the study site fed primarily on C3 plant‐based foods, which have lower δ^13^C values (mean δ^13^C = −29.8‰, *SD* = 1.6 ‰, *N *=* *17 species; Shaner et al., 2013) than the C4 sorghum (mean δ^13^C = −10.5‰, *SD* = 0.1‰, *N *=* *3 replicates; Shaner et al., 2017). Therefore, the higher a mouse's δ^13^C value, the more supplemental sorghum seeds it consumed. A blood sample (*c*. 100 μl) was taken from each nonpregnant, nonlactating adult mouse using retro‐orbital bleeding for isotopic analysis. No individuals were taken more than one blood sample within a month. Approximately, 1 mg of dried mouse plasma was placed into a tin cup, sealed, and transported to either the University of California Davis or the National Taiwan University for isotope measurements, which were performed on either a PDZ Europa 20–20 isotope ratio mass spectrometer (Sercon Ltd., Cheshire, UK) or a Thermo DELTA 5 isotope ratio mass spectrometer (Thermo Fisher Scientific Inc., Waltham, MA, USA). The mean percentage of sorghum seeds in mouse diets following food addition was estimated at 18% (range: 0%–31%; Table S2).

### 2.5 Parentage analysis

We conducted parentage analysis using 10 microsatellites (Hung et al. 2016). Ear tissue (~1.5 mm diameter) was collected from the mice using an ear punch. The tissue samples were submerged in 95% EtOH until DNA extraction. Genomic DNA was extracted from the ear tissues using the LiCl method, modified from Gemmell and Akiyama (1996). Polymerase chain reactions were performed following the protocols described in Hung et al. (2016). Amplicons were genotyped using ABI 3730XL sequencer, and allele sizes were scored using PeakScanner (Applied Biosystems). The program CERVUS 3.0 was used for parentage analysis (Kalinowski, Taper, & Marshall, 2007). The trio (both parents and the offspring) or pair (one parent and the offspring) confidence level was set at 80%.

### 2.6 Laboratory experiment on immune functions

Between October 2013 and January 2014, we captured 20 *A. semotus* (10 males, 10 females) from the study site for the laboratory experiment. Once brought back to the laboratory, they were housed individually in rodent cages (height: 16 cm, width: 25.5 cm, length: 47.3 cm). The total length of the experiment was 18 days. The mice were first acclimatized for 4 days, and on day 4, we randomly assigned half of the mice to a single dose of ivermectin (the other half received water). All mice were provided with 15 g of the same diets daily (Table S3). Given the small sample size, we chose to manipulate only parasite intensity rather than both food availability and parasite intensity because ivermectin treatment was safer to the mice than food deprivation. The mass‐specific energy intake per day (Kcal g^−1^ d^−1^) of each animal, averaged cross day 6–16, was calculated based on the consumption of each diet item, its energy content, and mouse body mass (Table S3). On day 9, we collected 0.05 g of feces to quantify FEC. Between day 16 and 18, we took 0.2 ml of blood for hematological analysis, which was performed at National Taiwan University (Haematology analyser, Medonic CA620, Spånga, Sweden). The hematological analysis quantified three metrics for immune functions, total white blood cell count (WBC), percent granulocyte count (GRAN), and percent lymphocyte count (LYMF).

### 2.7 Statistical analyses

The effects of sex, food, and parasitism on adult body mass, as well as offspring number and quality (i.e., mean body mass of all offspring; only offspring with body mass data as adults were included in this analysis) were tested using general or generalized linear models (normal distribution for adult body mass and offspring quality; Poisson distribution for offspring number). The fixed effects are sex, ivermectin treatment, δ^13^C values, and their interactions. We first tested all main and interaction effects, and then, we applied model selection approach to reduce the model complexity for final parameter estimates (Table S4). Because the ivermectin treatment reduced the infection intensity of the parasites rather than removed the parasites, the binary predictor of ivermectin treatment may not be able to reveal detailed relationships between parasite intensity, adult body mass and their reproductive performance. Therefore, we performed additional Spearman correlations between FEC and adult body mass, number of offspring, or quality of offspring. The potential trade‐off between number and quality of offspring was tested using Spearman correlation. To test whether degree of seed consumption alters parasite intensity, we performed Spearman correlation between FEC and δ^13^C values.

For the laboratory experiment on immune functions, we tested whether each of the three metrics (i.e., WBC, LYMF and GRAN) was affected by energy intake, FEC, and their interaction using general linear models. Because the ivermectin treatment did not affect these immune metrics in our preliminary tests (WBC: *F*_1,18_ = 0.38, *p *=* *.55; LYMF: *F*_1,18_ = 0.44, *p *=* *.52; GRAN: *F*_1,18_ = 2.14, *p *=* *.16) and considering the modest sample size, we pooled data across ivermectin‐treated and control mice.

All statistical analyses were performed in R 3.4.1.

### 2.8 Ethics note

The trapping and sampling of the mice were performed in accordance with the protocol approved by the Animal Care and Use Committee (protocol no. 102004) at the National Taiwan Normal University, Taipei, Taiwan.

## 3 RESULTS

### 3.1 Seed consumption and parasite intensity

The δ^13^C of a mouse was not correlated with its FEC, either for all mice combined (*p *=* *.53, *N* = 114) or for ivermectin‐treated and control females and males separately (treated females: *p *=* *.83, *N* = 15; control females: *p *=* *.86, *N* = 25; treated males: *p *=* *.87, *N* = 36; control males: *p *=* *.48, *N* = 38), suggesting that seed consumption did not alter parasite intensity.

### 3.2 Parentage assignment

A total of 285 individuals were subjected to parentage assignment. We were able to identify at least one parent for 42% of the individuals (both parents: 46, mother only: 37, farther only: 36). The success rate for the parentage assignment was modest, probably due to the fact that a portion of the 285 individuals tested was born to the parents that had died prior to the experiment as this species is capable of breeding year round. From the 46 offspring with both parents identified, we had five pairs of siblings from the same mothers who were captured as juveniles on the same day or on two consecutive days, suggesting they were from the same litter. Of the five pairs of siblings, four pairs had different fathers, indicating that *A. semotus* is promiscuous.

### 3.3 Adult body mass

Other than the known male‐biased size dimorphism in *A. semotus* (females = 24.6 g ± 0.57 *SE*; males = 27.6 g ± 0.37 *SE*), we did not find any effects of seed consumption or ivermectin treatment on adult body mass (Table 1 and Table S4). However, adult body mass was positively correlated with their FEC (*r*_*s*_ = .22, *p *=* *.01, *N* = 127), which was driven by the females (females: *r*_*s*_ = .32, *p *=* *.03, *N* = 47; males: *r*_*s*_ = .08, *p *=* *.48, *N* = 80).

**Table 1:** General or generalized linear models of adult body mass, offspring number, and offspring quality as a function of sex, food and parasitism in *Apodemus semotus*

Effect	Adult body mass (*N* = 128)				Offspring number (*N *= 146)				Offspring quality (*N* = 45)			
	Estimate	*SE*	*t*	*p*	Estimate	*SE*	*z*	*p*	Estimate	*SE*	*z*	*p*
Intercept	24.59	0.57	43.20	**<.0001**	3.55	0.98	3.63	**.0003**	7.22	7.42	0.97	.34
Sex	3.04	0.68	4.48	**<.0001**	**−**1.19	0.35	**−**3.35	**.001**	21.74	10.67	2.04	**.05**
δ^13^C	–	–	–	**–**	0.17	0.05	3.69	**.0002**	**−**0.82	0.33	**−**2.45	**.02**
Parasitism	–	–	–	–	**−**0.12	0.32	**−**0.36	.72	**–**	**–**	**–**	**–**
Sex × parasitism	–	–	–	–	0.87	0.45	1.94	**.05**	**–**	**–**	**–**	**–**
Sex × δ^13^C	–	–	–	–	**–**	**–**	**–**	–	0.98	0.50	1.95	.06

The food effect is the degree of seed consumption represented by the stable carbon isotopic value (δ^13^C) of mouse's plasma tissue. The parasitism effect is the ivermectin treatment (ivermectin vs. water). The sample sizes varied among models depending on data availability (i.e., adult body mass, δ^13^C values, offspring body mass measured during their adult stage). Significant effects are in bold. The model structures are determined through model selection (Table S3). The nonsignificant “parasitism” effect in the offspring‐number model is retained due to its interaction with “sex”. The marginal significance of “sex × δ^13^C” interaction in the offspring‐quality model is for parameter estimates; it was significant during model selection and therefore retained (Table S3).

Adult body mass was positively correlated with number of offspring (all: *r*_*s*_ = .17, *p *=* *.04, *N* = 153; females: *r*_*s*_ = .30, *p *=* *.03, *N* = 52; males: *r*_*s*_ = .18, *p *=* *.06, *N* = 101) but not quality of offspring (all: *p *=* *.91, *N* = 47; females: *p *=* *.40, *N* = 19; males: *p *=* *.54, *N* = 28), suggesting that the mice with higher body mass tended to produce more but not heavier offspring.

### 3.4 Reproductive performance

**Figure 1:**
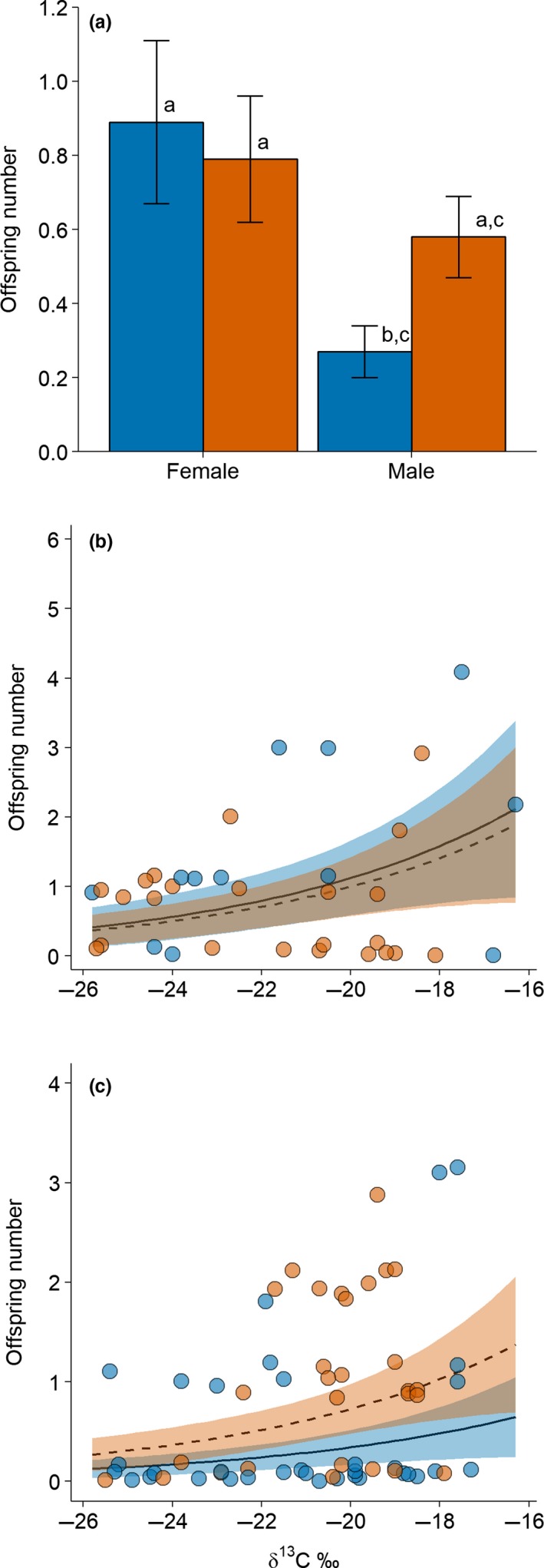
Number of offspring as a function of sex, ivermectin treatment, and seed consumption in *Apodemus semotus*. (a) All mice combined, (b) Female, (c) Male. The mean numbers of offspring for ivermectin‐treated (blue) and control (orange) females and males in (a) are estimated based on the final model in Table 1, with δ^13^C values fixed at the mean. The error bars denote 1 standard error. The degree of seed consumption increases with increasing stable carbon isotope values (δ^13^C) of mouse plasma tissue. The solid and dashed lines are the predicted means for ivermectin‐treated and control mice, respectively, with the shaded areas (blue shades for ivermectin‐treated mice; orange shades for control mice) indicating their 95% confidence intervals. The blue and orange circles are the actual observations for ivermectin‐treated and control mice, respectively. The offspring numbers are integers, but for visual display, a 20% vertical jittering was added

**Figure 2:**
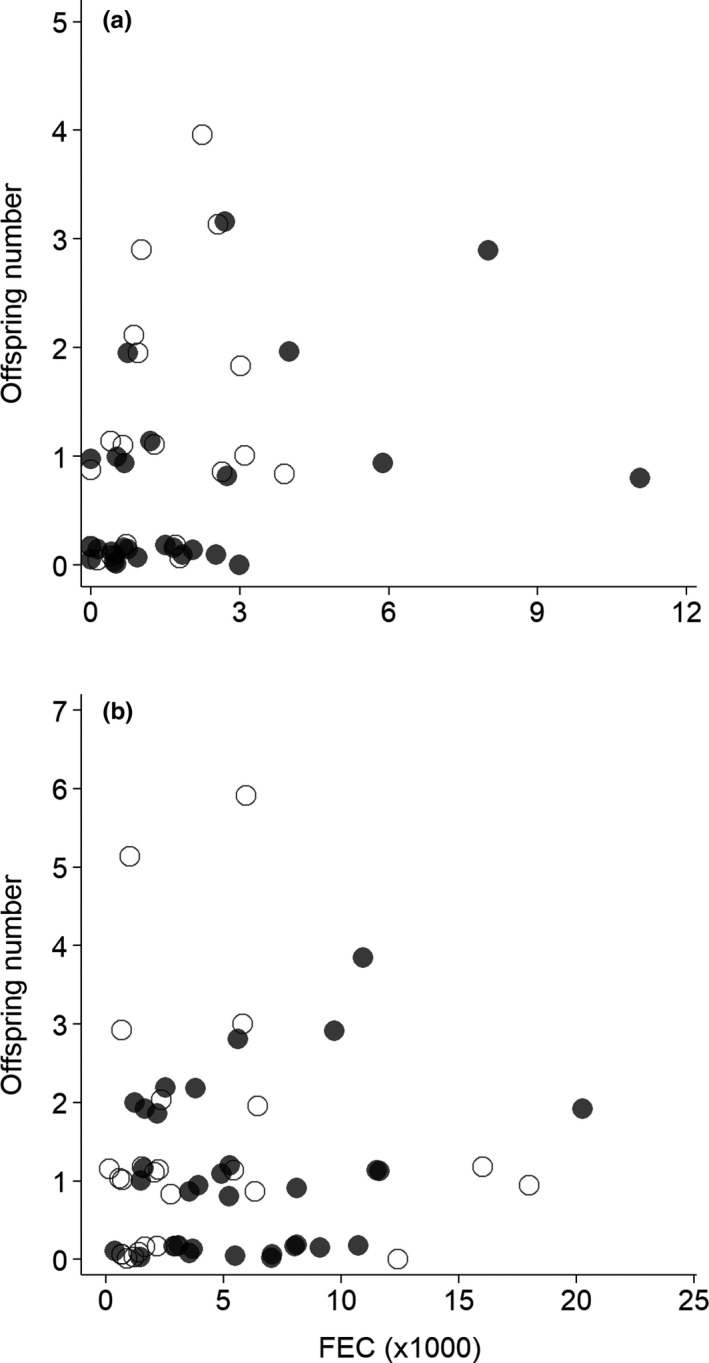
Correlations between parasite intensity and number of offspring in *Apodemus semotus*. (a) Ivermectin‐treated mice, (b) Control mice. The parasite intensity is quantified by the fecal egg count (FEC, number of nematode and cestode eggs per gram of mouse feces). The filled and unfilled circles denote the males and females, respectively. The offspring numbers are integers, but for visual display, a 20% vertical jittering was added

The females had a higher number of offspring than the males, driven by the lower number of offspring among ivermectin‐treated males compared to the females (Table 1; Figure 1a). A higher degree of seed consumption led to a higher number of offspring for all mice regardless of sex or ivermectin treatment (Table 1; Figure 1b and c). Although the ivermectin treatment did not affect the number of offspring (Table 1), the FEC and offspring number were positively correlated (*r*_*s*_ = .26, *p *=* *.001, *N* = 147), which was driven by ivermectin‐treated mice (treated females: *r*_*s*_ = .47, *p *=* *.01, *N* = 27; control females: *p *=* *.15, *N* = 36; treated males: *r*_*s*_ = .29, *p *=* *.06, *N* = 42; control males: *p *=* *.53, *N* = 42; Figure 2).

**Figure 3:**
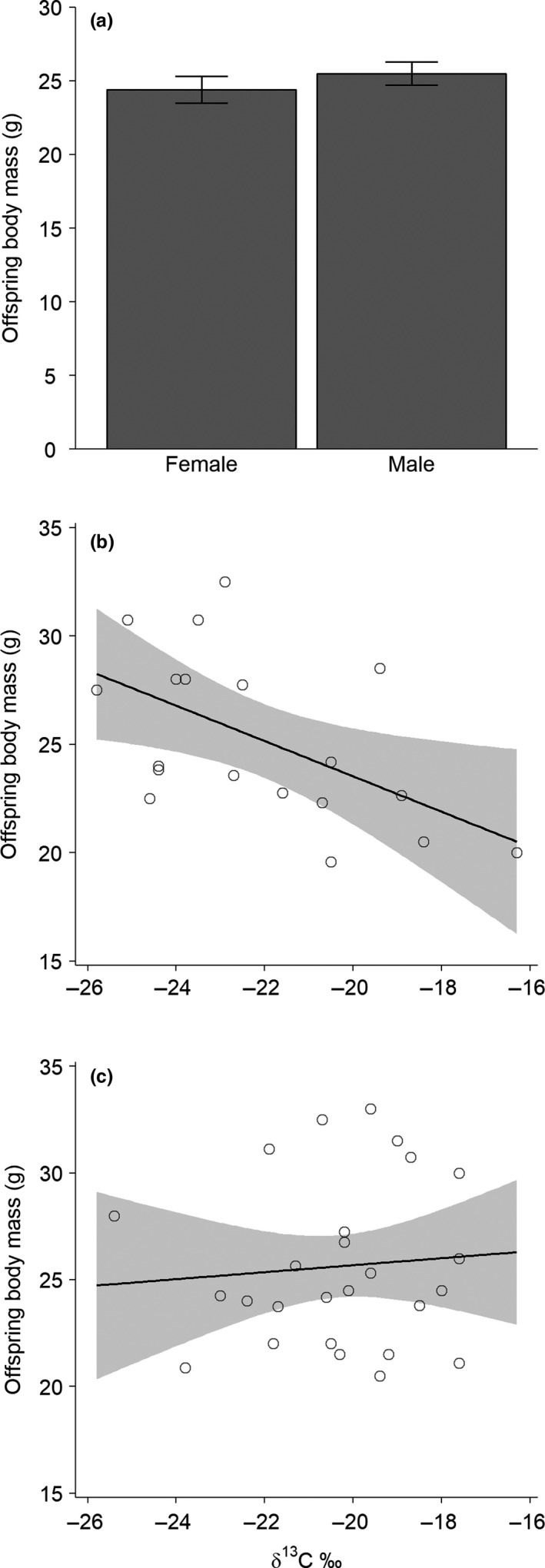
Quality of offspring as a function of sex and seed consumption in *Apodemus semotus*. (a) All mice combined, (b) Female, (c) Male. The mean body mass of offspring taken during their adult stage for the females and males in (a) are estimated based on the final model in Table 1, with δ^13^C values fixed at the mean. The error bars denote 1 standard error. The degree of seed consumption increases with increasing stable carbon isotope values (δ^13^C) of mouse plasma tissue. The solid lines are the predicted means, and the shaded areas are their 95% confidence intervals. The circles are the actual observations for individual mice

The offspring quality was not affected by ivermectin treatment (Table 1 and Table S4). The males had slightly higher quality offspring than the females (Table 1; Figure 3). Seed consumption had a negative effect on offspring quality for the females but not for the males (Table 1; Figure 3). The FEC and offspring quality were generally not correlated, except for the negative relationship among control males (all: *p *=* *.47, *N* = 53; ivermectin‐treated females: *p *=* *.70, *N* = 27; control females: *p *=* *.91, *N* = 15; ivermectin‐treated males: *p *=* *.21, *N* = 10; control males: *r*_*s*_ = −.64, *p *=* *.007, *N* = 16).

**Figure 4:**
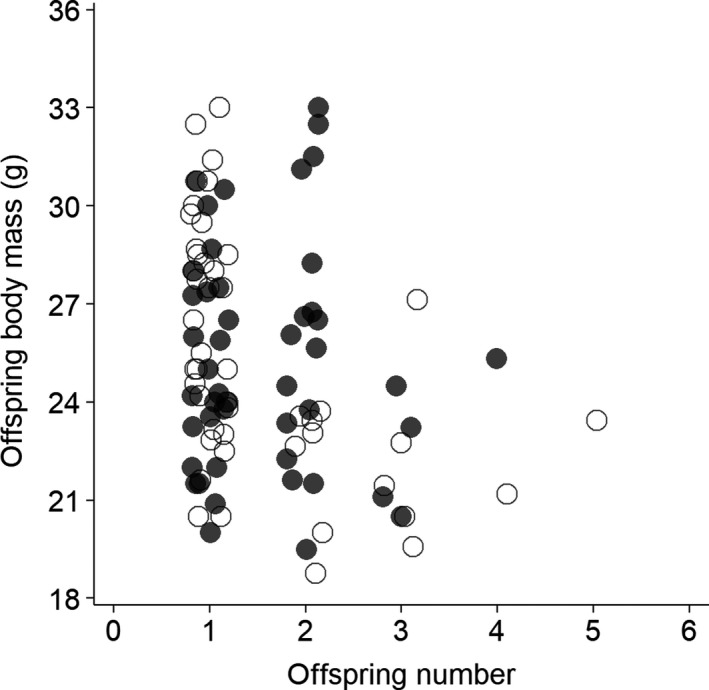
Relationship between number and quality of offspring in *Apodemus semotus*. The offspring quality is the mean body mass of all offspring for a given mouse, taken during the offspring's adult stage. The filled and unfilled circles denote the males and females, respectively. The offspring numbers are integers, but for visual display, a 20% horizontal jittering was added

The number and quality of offspring were negatively correlated for the females but not the males (all: *r*_*s*_ = −.33, *p *=* *.001, *N *= 95; females: *r*_*s*_ = −.58, *p *<* *.0001, *N* = 48; males: *r*_*s*_ = −.06, *p *=* *.70, *N* = 47; Figure 4), suggesting a female‐specific trade‐off between number and quality of offspring.

### 3.5 Immune functions, FEC, and energy intake

**Figure 5:**
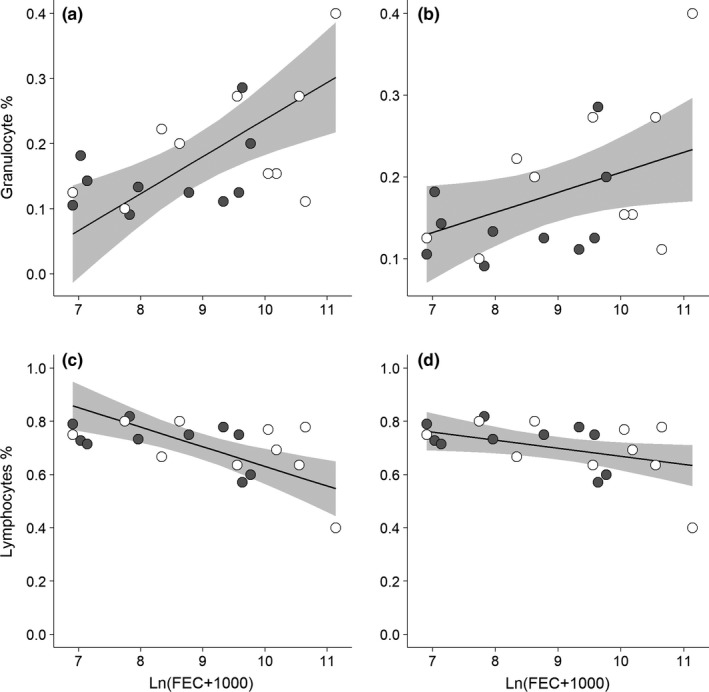
Predicted mean percent granulocyte (GRAN) and lymphocyte (LYMF) counts as a function of parasite intensity and energy intake in *Apodemus semotus*. (a) GRAN at low energy intake, (b) GRAN at high energy intake, (c) LYMF at low energy intake, (d) LYMF at high energy intake. Due to the significant interaction between energy intake and parasite intensity (FEC, number of nematode and cestode eggs per gram of feces), the predicted means are made with energy intake fixed at its first quantile of 0.43 kcal g^−1^ d^−1^ in (a) & (c), and at its third quantile of 0.54 kcal g^−1^ d^−1^ in (b) & (d). The solid lines are the predicted means and the shaded area their 95% confidence intervals. The filled and unfilled circles are actual observations for ivermectin‐treated and control mice, respectively

The energy intake and FEC did not affect WBC (Table 2). However, there were significant interactive effects of energy intake and FEC on GRAN and LYMF (Table 2). At a low level of energy intake, the FEC was positively associated with GRAN and negatively associated with LYMF. The relationships between FEC and GRAN or LYMF relaxed at a high level of energy intake (Figure 5), suggesting that energy intake could mediate the relationships between immune functions and parasite intensity.

**Table 2:** General linear models of immune functions as a function of energy intake and parasite intensity in *Apodemus semotus*

Effect	White blood cell count (10^3^/mm^3^)				Granulocyte %				Lymphocyte %			
	Estimate	*SE*	*t*	*p*	Estimate	*SE*	*t*	*p*	Estimate	*SE*	*t*	*p*
Intercept	6.62	27.26	0.24	.81	2.93	0.73	4.03	**.001**	−1.49	0.60	−2.49	**.02**
Energy intake	20.04	52.00	0.39	.70	−3.61	1.39	−2.61	**.02**	2.68	1.14	2.35	**.03**
FEC	0.34	3.04	0.11	.91	−0.24	0.08	−3.02	**.01**	0.19	0.07	2.79	**.01**
Energy intake × FEC	−2.00	5.78	−0.35	.73	0.40	0.15	2.57	**.02**	−0.30	0.13	−2.35	**.03**

The immune functions (i.e., total white blood cell count, percent granulocyte count, and percent lymphocyte count) are measured between day 16 and 18. The energy intake is mass‐specific (Kcal g^−1^ d^−1^) and averaged across day 6–16. The parasite intensity is represented by the fecal egg count (FEC, number of nematode and cestode eggs per gram of feces) measured on day 9. Significant effects are in bold.

## 4 DISCUSSION

Food availability had positive effects on the reproductive performance of *A. semotus*, which is common for small mammals (reviewed in Bronson, 1985; Bronson & Perrigo, 1987; Boutin, 1990; Speakman, 2008). In the field experiment, we found that a higher degree of seed consumption increased the number of offspring in *A. semotus*. However, for the females, there was also a trade‐off between number and quality of offspring. Consequently, with increasing seed consumption, the females produced more offspring of lower quality. This female‐specific trade‐off suggests that the higher energetic demands of gestation and lactation make food availability an important factor of female reproductive performance.

Parasitism of intestinal nematodes and cestodes likely has a neutral effect on *A. semotus* reproduction. The ivermectin treatment did not affect the number and quality of offspring in *A. semotus*. The number of offspring increased with parasite intensity (FEC) among ivermectin‐treated mice but not control mice, suggesting that the treated mice were retaining or regaining infection intensity as they produced more offspring. Furthermore, adult body mass was positively correlated with both number of offspring and FEC, suggesting that larger mice may be able to tolerate a higher parasite intensity without reducing their numbers of offspring. Between sexes, the females had clearer patterns in the positive associations between parasite intensity and number of offspring, parasite intensity and adult body mass, as well as adult body mass and number of offspring, suggesting that the females may trade parasitism defenses for reproduction via increased parasite tolerance. The only potential negative effect of parasitism on *A. semotus* reproduction was found for the control males in our field experiment, although this is based on a small sample size. The quality of offspring and parasite intensity was negatively correlated among control males. Unlike the females, the males did not exhibit a trade‐off between number and quality of offspring. Moreover, unlike the treated males, the control males did not have an increased number of offspring with increasing parasite intensity. Therefore, a high parasite intensity could reduce male reproductive performance under natural conditions.

Many studies have shown negative effects of parasitism on small mammal reproduction (Gooderham & Schulte‐Hostedde, 2011; Neuhaus, 2003; Patterson & Schulte‐Hostedde, 2011; Patterson et al., 2013). In fact, a recent study on *A. semotus* (Lo & Shaner, 2015) found that less‐parasitized females had a higher maternal investment, suggesting negative effects of parasitism on their reproduction. However, these studies focused on reproductive performance from a single breeding season for species that could breed across several years (e.g., squirrels, chipmunks) or a single breeding event for species that could breed multiple times within a breeding season (e.g., *A. semotus*), which can be different from their lifetime reproduction. The life span of *A. semotus* is typically <1 year, and our study period included their peak breeding season (Lin et al., 2014; Lo & Shaner, 2015). Therefore, the number and quality of offspring in this study likely reflected their lifetime reproductive performance. Our results indicated that, despite the negative effects of parasite intensity on the maternal investment of *A. semotus* during a single breeding event, parasitism might be neutral to their lifetime reproductive success.

The natural prevalence of intestinal nematodes and cestodes is high for *A. semotus* (70%–100%; Lo & Shaner, 2015; Shaner et al., 2017; this study). Therefore, it is logistically difficult to reduce parasite intensity to a near‐zero level. Nevertheless, the ivermectin treatment in our field experiment had led to an average of 68%–69% reduction in FEC for the treated females and males compared to the control. In another study on *A. semotus*, the FEC was reduced by *c*. 73% using ivermectin, and the treated females were found to increase their maternal investment during a single breeding event (Lo & Shaner, 2015), suggesting that the ivermectin treatment in our field experiment was likely sufficient in reducing parasite intensity to a level relevant to host reproduction. We estimated that individual *A. semotus* in the field experiment incorporated on average 18% of the supplemental seeds in their diets. Compared to *c*. 68%–69% reduction in FEC by the ivermectin treatment, the food addition was not necessarily more effective than the ivermectin treatment yet it had significant impacts on *A. semotus* reproduction. Therefore, the lack of parasitism effects on *A. semotus* reproduction was not likely explained by lower treatment effectiveness.

In the field experiment, we did not find any interactive effects of food addition and ivermectin treatment on *A. semotus* reproduction. However, in the laboratory experiment, the percentages of lymphocyte and granulocyte counts were associated with FEC only for the mice with a low energy intake but not for those with a high energy intake. These observations suggest that an increased food availability may facilitate a switch from immuno‐defenses of parasites to parasite tolerance (i.e., relaxed immuno‐defenses) in *A. semotus*. In boreal Europe, field voles with high food availability mounted stronger immune responses against nematode infections than food‐limited voles (Forbes et al., 2016). There are several explanations for why we detected relaxed immuno‐defenses with increasing food availability rather than stronger immune responses as previously reported in Forbes et al. (2016). First, our system is in a subtropical montane forest where food may not be as limiting as in boreal Europe, and therefore food availability is not likely to constrain overall levels of immune responses. Second, the results from Forbes et al. (2016) were based on a field enclosure study, whereas ours were based on a laboratory experiment where the conditions were generally less harsh than in field. Finally, the probability of parasitic infection was likely much higher in our system (the prevalence of intestinal nematodes was <40% in Forbes et al., 2016 and *>*90% in our system; also see Shaner et al., 2017), which could make tolerance a better strategy than immuno‐defenses. The role of food availability in modulating host immune responses is complex, and different patterns likely exist among systems with various background levels of food availability and infection risks.

In our field experiment, assuming the food stations attracted more mice and therefore had more feces in the areas, the mice who consumed more supplemental seeds that allowed them to produce more offspring could also be experiencing higher infection risks due to their close proximity to the food stations. Such spacing behaviors may help explain the positive associations between parasite intensity and number of offspring found in our field experiment. However, we did not find the mice to be spatially aggregated at the food stations (Figure S3), which is consistent with their typical spacing behaviors (e.g., high site fidelity, stable home ranges, limited mobility; Lin & Shiraishi, 1992b; Shaner et al., 2017). Furthermore, the infection risks (i.e., the mean FEC of all mouse fecal samples taken at a given trapping station) for the entire study site had been previously reported in Shaner et al. (2017). They found that the infection risks tended to be lower, not higher, in the areas close to the food stations. Therefore, the spacing behaviors of *A. semotus* were not likely to fully account for the positive associations between parasite intensity and number of offspring.

This study adds to the increasing evidence for positive effects of food availability on small mammal reproduction. With increasing food availability, we found that individual *A. semotus* produced more offspring. However, the fitness benefits of food availability might be different between sexes given the female‐specific trade‐off between number and quality of offspring. The effects of intestinal parasites on *A. semotus* reproduction were less clear. Except for a potential negative effect on offspring quality for the males, the number of offspring in *A. semotus* generally increased with increasing parasite intensity, suggesting that they might be able to tolerate parasitic infection to a degree without reducing number of offspring. We suggest that host individuals may use parasite tolerance rather than immuno‐defenses as a way to cope with competing energetic demands of reproduction and parasitic infection, in a system where parasite prevalence is high and food availability is not limiting, such as *A. semotus* in this study.

## CONFLICT OF INTEREST

None declared.

## AUTHORS’ CONTRIBUTIONS

PLS conceived and designed the experiment, performed the statistical analyses, and led the writing of the manuscript. PLS, AYY, and CHH collected the field data. AYY and SHL performed the paternity analysis. CHH performed the laboratory experiment. All authors contributed critically to the drafts and gave final approval for publication.

## DATA ACCESSIBILITY

Data available from the Dryad Digital Repository https://doi.org/10.5061/dryad.6545f86
