# Supplementary material for: The effects of food and parasitism on reproductive performance of a wild rodent
Source: Ecol Evol. 2018 Mar 26;8(8):4162–72. doi: 10.1002/ece3.3997 (PMC5916304; doi:10.1002/ece3.3997)

**Supporting Information for**

**The effects of food and parasitism on reproductive performance of a wild rodent**

**Pei-Jen L. Shaner, Ai-Yun Yu, Shou-Hsien Li,** Ching-Ho Hou

Department of Life Science, National Taiwan Normal University, Taipei, Taiwan

Corresponding author: Pei-Jen L. Shaner ([pshaner@ntnu.edu.tw](mailto:pshaner@ntnu.edu.tw))

*Running title:* Food, parasitism and rodent reproduction

**Table S1. Generalized linear model for the faecal egg count of intestinal nematodes and cestodes in *Apodemus semotus* as a function of the ivermectin treatment and sex.** The faecal egg count (FEC) is the number of eggs from strongyle nematodes, *Strongyloides* spp., *Trichuris* spp., *Syphacia* spp., *Physaloptera* spp., *Ascaris* spp., *Heterakis* spp. and *Hymenolepis* spp. per gram of mouse faeces. The pre-treatment FEC is based on the faecal sample taken at the time when an individual first received the ivermectin/water treatment, and is included as a covariate. The model is fitted to negative binomial distribution. The data set includes 142 adult mice (58 females, 84 males), all with pre- and post-treatment FEC values. Only the mice with a none-zero pre-treatment FEC (i.e. originally infected with intestinal parasites) were included in this analysis of ivermectin efficacy. Significant effects are in bold.

| Effect | Estimate | SE | *z* | *P* |
| --- | --- | --- | --- | --- |
| Intercept | 7.01 | 0.24 | 29.24 | **<0.0001** |
| Sex | 0.35 | 0.28 | 1.24 | 0.22 |
| Ivermectin | 1.13 | 0.30 | 3.74 | **0.0002** |
| Sex × ivermectin | 0.04 | 0.39 | 0.11 | 0.91 |
| Pre-treatment FEC | 0.00 | 0.00 | 1.66 | 0.10 |

**Table S2. Estimated amount of consumption of the sorghum (*Sorghum bicolour*) seeds by *Apodemus semotus* in the field experiment.** The calculations are based on the mass balance equation ([δ^13^C_post-addition_ - δ^13^C_pre-addition_] / [δ^13^C_sorghum-seeds_ - δ^13^C_C3-plants_]) with the parameter values of δ^13^C_C3-plants_ = −29.8‰ (i.e. mean values of 17 most common C3 plants at the study site; Shaner et al., 2013) and δ^13^C_sorghum-seeds_ = −10.5‰; Shaner et al., 2017). Because the supplemental seeds were put out early during the experiment, we did not have a large number of mice with pre-addition blood samples. Therefore, this calculation was done using the data on 24 individuals with both pre- and post-addition samples. The mean values (±SD) of δ^13^C_post-addition_ and δ^13^C_pre-addition_ for each of the 4 sex × ivermectin treatment group, as well as all 24 individuals combined, were provided for reference. The mean percentage of sorghum seeds in mouse diets (±SD), however, is calculated based on individual values of δ^13^C_post-addition_ and δ^13^C_pre-addition_. The ranges of percentage of sorghum seeds in mouse diets are provided in parentheses.

| Sex | Ivermectin treatment | N | δ^13^C_post-addition_ (‰) | δ^13^C_pre-addition_ (‰) | Percentage of sorghum in diet (%) |
| --- | --- | --- | --- | --- | --- |
| Female | Ivermectin | 1 | -20.5 | -23.1 | 13 |
|  | Control | 4 | -21.2±2.8 | -23.8±0.8 | 14±12 (0-30) |
| Male | Ivermectin | 11 | -20.6±2.6 | -23.7±0.4 | 17±13 (0-31) |
|  | Control | 8 | -19.8±1.2 | -24.0±0.5 | 22±6 (11-30) |
| All |  | 24 | -20.4±2.2 | -23.8±0.5 | 18±10 (0-31) |

Shaner, P. L., Wu, S. H., Ke, L., & Kao, S. J. (2013). Tropic niche divergence reduces survival in an omnivorous rodent. Evolutionary Ecology Research, 15, 1-14.

Shaner, P. L., Yu, A. Y., Ke, L., & Li, S. H. (2017). Spacing behaviors and spatial recruitment of a wild rodent in response to parasitism. Ecosphere, 8, e01780.

**Table S3. Diet provided to *Apodemus semotus* in the laboratory experiment.** The diet items with superscriptions a, b are provided on alternate days. The mealworms and super worms are larva of *Tenebrio molitor*.

| Diet | Energy (Kcal/100g) | Protein (%) | Water content (%) | Daily provision (g) |
| --- | --- | --- | --- | --- |
| Corn^a^ | 111 | 3.8 | 74.2 | 2 |
| Pumpkin^b^ (fruit) | 64 | 2.4 | 82.3 | 2 |
| Mealworm^a^ | 196 | 20.3 | 62.4 | 1 |
| Superworm^b^ | 231 | 17.4 | 59.4 | 1 |
| Potato | 124 | 1 | 69.2 | 6 |
| Green bean | 307 | 22.1 | 0 | 1.2 |
| Rice | 354 | 7.2 | 0 | 1.2 |
| Sunflower (seed) | 584 | 21 | 0 | 1.2 |
| Oat | 389 | 16.9 | 0 | 1.2 |
| Barley | 357 | 7.2 | 0 | 1.2 |

**Table S4. Model selection for adult body mass, offspring number and offspring quality in *Apodemus semotus*.** The food effect is the degree of seed consumption represented by the stable carbon isotope value (δ^13^C) of a mouse’s plasma tissue. The parasitism effect is the ivermectin treatment. Each predictor, from the three-way to two-way interactions, and main effects that are not involved in the interactions already retained, is removed in turn. The change in model fit (RSS, residual sums of squares) between a reduced model and the model it immediately nested in is assessed with a χ^2^ test for offspring number (generalized linear models with Poisson distribution) or a *F* test for adult body mass and offspring quality (general linear models). Significant reductions in RSS are indicated by star signs next to the statistics (*: <0.05; **: <0.01; ***: <0.001; ****: <0.0001).

| Model | Adult body mass (N = 128) | | Offspring number (N = 146) | | Offspring quality (N = 45) | |
| --- | --- | --- | --- | --- | --- | --- |
|  | RSS | *F* | RSS | χ^2^ | RSS | *F* |
| Sex + δ^13^C + parasitism + sex × δ^13^C + sex × parasitism + δ^13^C × parasitism  + sex × δ^13^C × parasitism | 1503.6 | - | 176.21 | - | 524.90 | - |
| Sex + δ^13^C + parasitism + sex × δ^13^C + sex × parasitism + δ^13^C × parasitism | 1530.5 | 2.15 | 176.88 | 0.68 | 524.92 | 0.001 |
| Sex + δ^13^C + parasitism + sex × δ^13^C + sex × parasitism | 1536.2 | 0.45 | 177.08 | 0.20 | 547.04 | 1.60 |
| Sex + δ^13^C + parasitism + sex × δ^13^C + δ^13^C × parasitism | 1533.0 | 0.20 | 181.09 | 4.21* | 524.98 | 0.004 |
| Sex + δ^13^C + parasitism + sex × parasitism + δ^13^C × parasitism | 1531.6 | 0.08 | 178.41 | 1.53 | 593.74 | 4.98* |
| **Sex + δ^13^C + parasitism + sex × parasitism (final model: offspring number)** | - | - | 178.49 | - | - | - |
| Sex + δ^13^C + parasitism + sex × δ^13^C | - | - | - | - | 552.39 | - |
| **Sex + δ^13^C + sex × δ^13^C (final model: offspring quality)** | - | - | - | - | 553.11 | 0.05 |
| Sex + δ^13^C + parasitism | 1538.7 | - | - | - | - | - |
| Sex + δ^13^C | 1543.8 | 0.41 | - | - | - | - |
| Sex + parasitism | 1545.4 | 0.54 | - | - | - | - |
| δ^13^C + parasitism | 1797.5 | 20.86**** | - | - | - | - |
| **Sex (final model: adult body mass)** | 1551.1 | - | - | - | - | - |

**Fig. S1. Map of the study site.** The circles indicate the locations of the trapping stations. The squares indicate the locations of the trapping stations used as food stations.

**
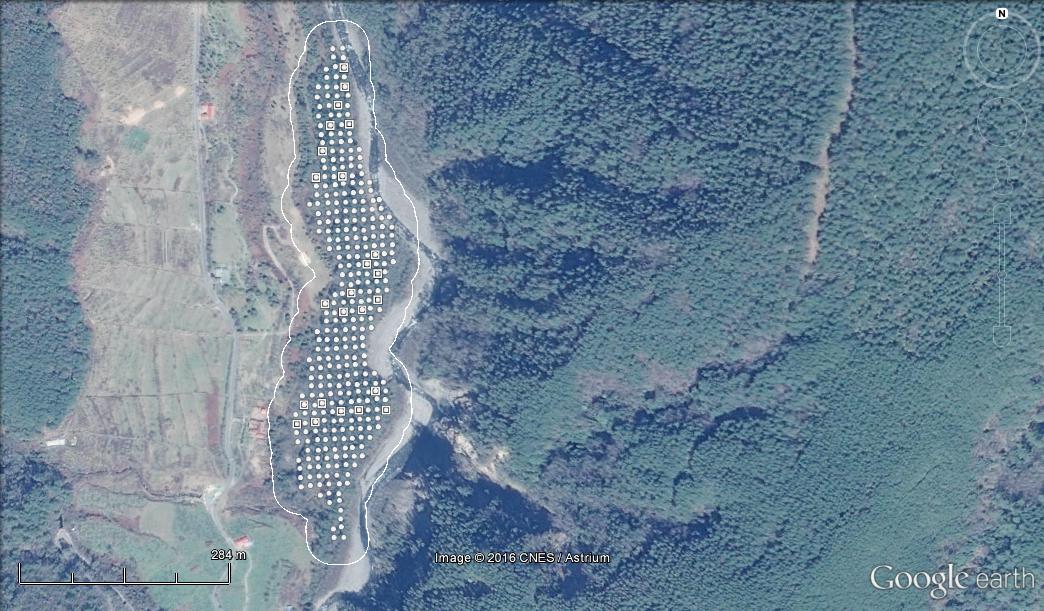
**

**Fig. S2. Effects of the ivermectin treatment on the faecal egg count of intestinal nematodes and cestodes in *Apodemus semotus*.** The faecal egg count (FEC) is the number of eggs from strongyle nematodes, *Strongyloides* spp., *Trichuris* spp., *Syphacia* spp., *Physaloptera* spp., *Ascaris* spp., *Heterakis* spp. and *Hymenolepis* spp. per gram of mouse faeces. The blue and orange bars denote the ivermectin-treated and control mice respectively. The error bars are one standard error.


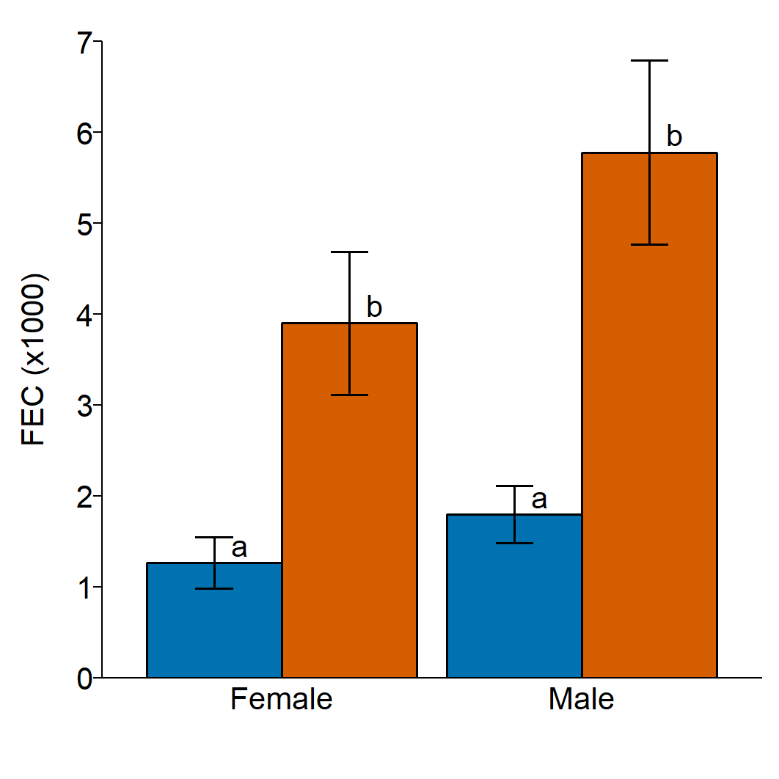


**Fig. S3. Frequency distribution of bootstrapped mean numbers of *Apodemus semotus* captures.** Each bootstrapped mean number of captures is based on the mean of 24 randomly selected trapping stations. The actual mean number of *A. semotus* captures at the 24 food stations is indicated by the triangle. Compared to the 1000 bootstrapped replicates under the assumption of random space use by the mice, the actual mean number of captures at the food stations is ranked at 850, suggesting no statistical difference between the food stations and randomly-selected trapping stations (*P* = 0.15).


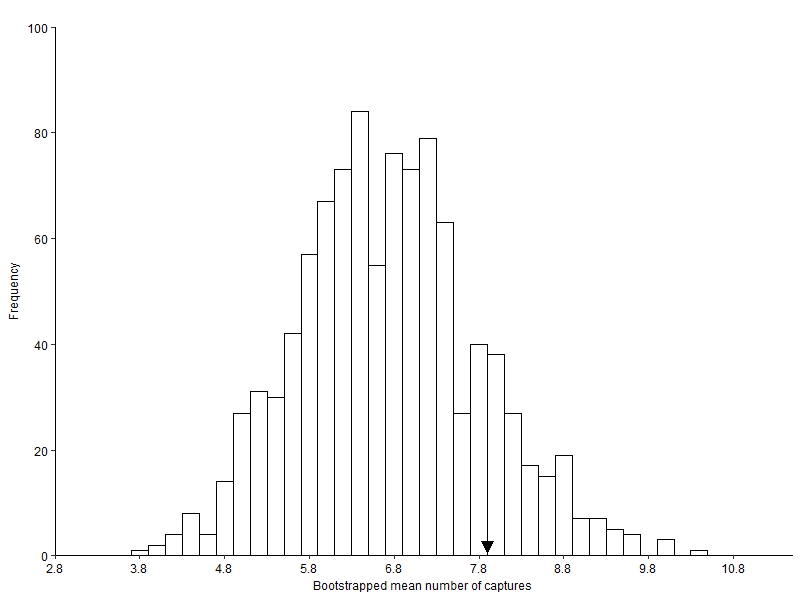

Supplement: Supplementary file 1 [file ECE3-8-4162-s001.docx]
